# Post-Exercise Recovery Modalities in Male and Female Soccer Players of All Ages and Competitive Levels: A Systematic Review

**DOI:** 10.3390/sports13100343

**Published:** 2025-10-02

**Authors:** Emaly Vatne, Jose M. Oliva-Lozano, Catherine Saenz, Rick Cost, Josh Hagen

**Affiliations:** 1 Human Performance Collaborative, The Ohio State University, Columbus, OH 43221, USA; 2 United States Soccer Federation, Chicago, IL 60604, USA; 3 College of Education and Human Ecology, The Ohio State University, Columbus, OH 43221, USA; saenz.11@osu.edu

**Keywords:** soccer, recovery, cold-water immersion

## Abstract

Optimal recovery supports health and enhances performance in soccer players, yet the empirical evidence on various recovery strategies in soccer is complex to interpret. This review aimed to summarize the literature on post-exercise recovery modalities in male and female soccer players of all ages and competition levels. Following PRISMA guidelines, PubMed, SPORTDiscuss, and Web of Science were systematically searched until 17 October 2023. Randomized controlled trials or within-subjects crossover design studies that examined the effects of post-exercise recovery interventions on physical, psychological, or performance outcomes in soccer players were included. A single reviewer extracted data and assessed study quality using the Physiotherapy Evidence Database (PEDro) scale. Overall, 41 studies were included in the final review. The recovery strategies represented in these studies were organized into the following categories: active recovery, blood flow restriction, cold water immersion, contrast water therapy, compression garments, active cool-down, cryotherapy, cold garments, sleep and daytime nap, pneumatic cooling, foam rolling, mindfulness interventions, nutritional intervention, and static stretching. The findings demonstrated that cold-water immersion consistently improved jump performance and perceptions of fatigue, soreness, and overall well-being. Other recovery strategies, such as active recovery, compression therapy, sleep interventions, and nutrition supplementation, also positively impacted recovery, albeit with varying levels of effectiveness and evidence. However, the studies exhibited heterogeneity in methods, outcome measures, and recovery intervention protocols, posing challenges for generalizability. This review summarizes recovery strategies for soccer players, emphasizing the need for practitioners, coaches, and athletes to individualize interventions based on athletes’ needs, preferences, and competition level.

## 1. Introduction

Timely recovery is crucial for achieving optimal athletic performance [1,2]. Recovery is a broad construct, but it can be operationally defined as the restoration of an athlete’s ability to meet or exceed the previous psychophysiological state or performance in a particular activity [3,4]. While training and competition place acute physical and psychological stressors on athletes, it is the time outside of training when the improvements in psychophysiological capacities develop [3,4]. Failing to recover fully may result in the athlete being predisposed to an increased risk of injuries and reduced capacity to meet the volume or intensity demands of the sport [5]. Thus, maximizing and accelerating responses to training and competition demands is critical to supporting the health and athletic performance of athletes.

Soccer is a high-intensity, team sport, with an intermittent activity profile [6]. Optimal and personalized recovery is essential to maintain health, support athletic development, and improve athletic performance in soccer players. This is in part due to the sport’s combined anaerobic and aerobic metabolic demands, large neuromuscular load, and high-level performance demands (i.e., changes of direction, accelerations, decelerations, and high- and low-intensity actions) [7,8,9,10,11,12]. The physicality of a soccer match can be observed at every competition level, affecting both male and female athletes. For example, for as long as 48–72 h after a match, professional-level male soccer athletes reported a perceived impaired recovery state [12], fatigue [13], and delayed onset muscle soreness [14]. Similarly, elite female soccer athletes presented with reduced physical capacity and increased soreness for up to 69 h (about 3 days) following match participation [15]. In conjunction with changes in subjective perceptions of fatigue and soreness, changes in physiological biomarkers like creatine kinase, cortisol, and interleukin-6 also characterize muscle damage, stress, and inflammation, respectively, following male and female soccer matches [15,16]. Matches also affect neuromuscular performance and psychological wellness [17]. Neuromuscular fatigue, identified through countermovement jump performance, can be present 48 to 72 h following a match in elite soccer players [15].

Soccer players commonly use a spectrum of recovery strategies to accelerate their responses to, and recovery from, training and competition demands [3,18]. The purpose of supplemental recovery strategies is to accelerate an athlete’s shift away from stress and toward recovery on the stress–recovery continuum, encouraging an athlete to recover faster [4]. The use of appropriate recovery strategies can lead to improved athletic performance and athletes feeling healthier and more rested [19]. However, recovery prescriptions must be specific to the needs of an individual athlete and informed by empirical evidence, but the research describing the effects of highly utilized recovery strategies is lacking, especially in soccer players [20]. A systematic review of the effects of recovery modalities in elite male soccer players recently highlighted what is known about how these strategies support athletes’ responses to training and matches, but the review only included seven studies [20]. Furthermore, by only including professional male soccer players, this review may have overlooked how female soccer players and soccer players of different ages and competitive levels may respond to the use of recovery strategies [20].

Athletes, coaches, and practitioners need to understand the effects and underlying rationale for each recovery strategy in the environment in which it is being applied [5]. However, research describing the effects of recovery interventions in soccer players of various ages and competition levels can be challenging to navigate and contextualize. Therefore, the primary aim of this study was to systematically review the literature on the effects of post-exercise recovery modalities in male and female soccer players of all ages and competitive levels using randomized controlled trials.

## 2. Materials and Methods

The methods of this systematic review were informed by Preferred Reporting Items for Systematic Reviews and Meta-Analysis (PRISMA) guidelines [21,22]. A literature search was conducted across three electronic databases (PubMed, SPORTDiscus, and Web of Science) up until 17 October 2023. The title and abstract search criteria are presented in Table 1. Boolean logic operators were used such that a sample of soccer athletes and a recovery modality or recovery modalities were combined with any measure of psychophysiological recovery or performance outcomes, i.e., (1 AND 2) AND (3 OR 4 OR 5).

Search results were compiled in Microsoft Excel (Version 2308, Microsoft Excel, Redmond, WA, USA), and article duplicates were removed. Next, titles then abstracts were screened for relevance and initial eligibility. Full-text manuscripts of the studies that were retained after the initial screening were independently assessed against the inclusion and exclusion criteria by two independent reviewers. Reference lists of the full-text articles were also screened for relevant titles that may have been missed in the initial electronic database search.

Studies were included if they met all of the following criteria: (1) participants were members of a soccer team at the time of the study, (2) deployed a post-exercise (immediately post-training or post-competition) protocol that included the use of a recovery modality, (3) randomized controlled trial or randomized controlled, within-subjects cross-over designs, (4) reported a psychophysiological or performance outcome variable, (5) written in English, and (6) was published in a peer-reviewed journal. Studies were excluded if they were a literature review, case study, commentary, opinion article without original data, gray literature, or if the study participants included injured athletes.

Study characteristics, participant information, recovery intervention context, and outcomes were extracted for the included full-text manuscripts that were retained after screening against inclusion and exclusion criteria by a single reviewer. While a single reviewer screened the full-text manuscripts, any uncertainties were resolved through discussion with a co-author. The spreadsheet used to compile the extracted data followed the template published by the Cochrane Consumers and Communication Review Group [23]. Extracted information included authors, year of publication, sample characteristics (e.g., age, gender, and competitive level), study design, recovery modality, and name, recovery protocol, control group protocol, fatiguing exercise, assessment timepoints, and main findings.

The full-text manuscripts that were included in this final review were screened for their methodological quality using the Physiotherapy Evidence Database (PEDro) scale. The PEDro scale consists of 11 items describing eligibility criteria, random allocation, concealed allocation, group similarity at baseline, subject, administrator, and assessor blinding, >85% completion, intention-to-treat analysis, between-group comparison for at least one key outcome, and point and variability measures for at least one key outcome. The maximum score was 11 points (poor quality: 0–3; fair quality: 4–6; good quality: 6–8; excellent quality: 9–11) [24]. Ambiguous characteristics or scoring of a study were resolved by consensus between two independent researchers.

## 3. Results

### 3.1. Search Results

**Figure 1:**
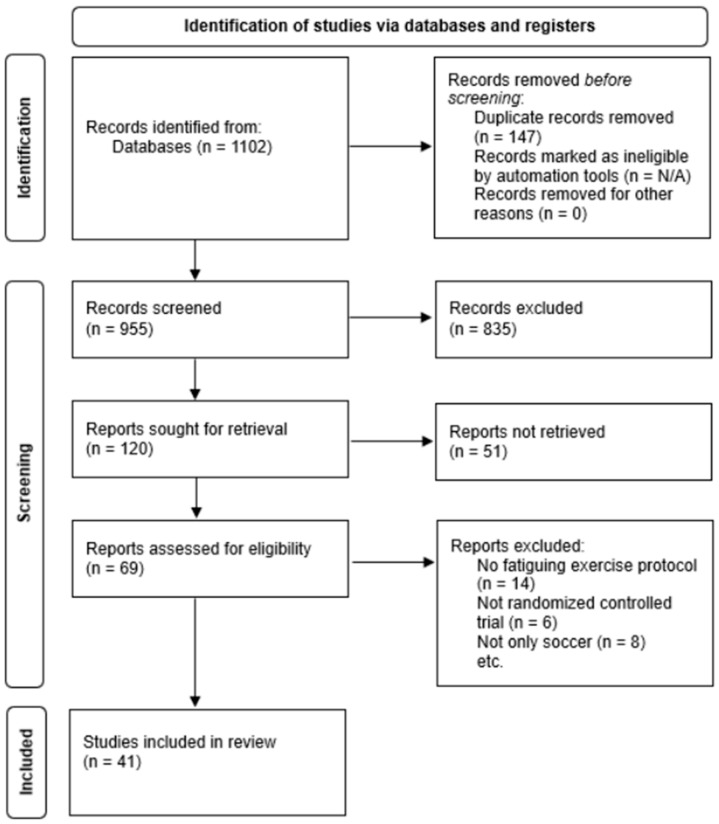
Selection process of the included studies using the Preferred Reporting Items for Systematic Reviews and Meta-Analyses (PRISMA) flowchart.

The initial search identified 1102 references, of which 120 studies remained for analysis after duplicates were removed, and titles and abstracts were screened for relevance against the inclusion criteria. Following the evaluation of the full texts, 41 studies were included in the final review (Figure 1).

### 3.2. Study Characteristics and Quality Assessment

The characteristics of the studies found in this review are presented in Tables S1–S4 in the Supplementary Materials. When combining all the studies, a total of 685 soccer players were included in this review, of which 630 were male (91.97%) and 55 (8.03%) were female. Competition levels included youth (*n* = 200, 29.19%), collegiate/non-professional (*n* = 125, 18.24%), semi-professional (*n* = 124, 18.18%), professional (*n* = 226, 32.99%), and not described (*n* = 10, 1.4%). Sample sizes ranged from 8 to 40, and the mean age of the participants was between ~14.3 and ~25.4 years old.

The methodological quality assessment reflected that the studies selected for this review were generally of fair quality, considering the average PEDro score was ~6.8 (range: 3–10) out of 11 possible points (Table 2).

### 3.3. Recovery Strategies

The recovery strategies in the identified studies focused on: active recovery (*n* = 6) [15,31,52,54,55,56], blood flow restriction (*n* = 2) [35,38], cold-water immersion (*n* = 12) [28,32,33,34,37,44,46,51,52,59,60,61], contrast-water therapy (*n* = 1) [45], compression garments (*n* = 3) [28,49,50], active cool-down (*n* = 2) [48,58], whole-body cryotherapy chamber (*n* = 2) [40,62], cold garments (*n* = 1) [36], pneumatic cooling (*n* = 1) [29], foam rolling (*n* = 1) [57], mindfulness interventions (*n* = 1) [64], nutritional intervention (*n* = 7) [25,26,27,30,39,42,47], sleep and daytime nap (*n* = 3) [41,43,63], and static stretching (*n* = 1) [53]. These were further organized into the following subsections for further assessment: hydrotherapy, active recovery and cool-down, blood flow restriction and compression therapy, muscle relaxation and flexibility, whole-body cryotherapy and cooling garments, mindfulness, sleep, daytime nap, and nutrition.

### 3.4. Hydrotherapy

Regarding hydrotherapy, jump test (e.g., squat jump, countermovement jump) performance [33,34,46,51] and perceived soreness, fatigue, and overall well-being [32,45,52,59] were most frequently improved (8 of 13 hydrotherapy studies). Attenuated post-exercise jump performance impairments coupled with reductions in perceived soreness were found following cold-water immersion in semi-professional male soccer players [33,51]. Similar findings of minimized soreness and accelerated pain reduction following the use of post-exercise cold-water immersion were also reported in studies with academy and semi-professional male soccer players, but no effects of cold-water immersion on maximal voluntary isometric contraction or postural control were observed in these populations [32,45,52].

The impact of this cold-water immersion on blood biomarkers was inconsistent across studies. Creatine kinase was not different following 10 min of post-exercise cold-water immersion at 10 °C compared to thermoneutral-water immersion in semi-professional male soccer players [33] or following 20 min of post-exercise cold-water immersion at 10 °C in male professional soccer players [46]. Two studies revealed attenuated impairments of creatine kinase following 10 min of cold-water immersion at 10 °C after fatiguing exercise tests compared to thermoneutral water immersion in professional and youth male soccer players [32,34].

### 3.5. Active Recovery and Cool-Down

Three studies revealed a beneficial effect of active recovery and cool-down on countermovement jump performance and perceived pain and soreness, but not on blood biomarkers or session rating of perceived exertion. The recovery benefits were observed in studies including male professional and club soccer players [54,55,56], but not in the two studies consisting of professional and semi-professional female soccer players [15,52]. No study reported a significant effect of active recovery on blood biomarkers [15,31,54]. The biomarkers that were analyzed but found to be unaffected by active recovery included muscle damage and/or oxidative stress markers such as creatine kinase, urea, reduced glutathione, oxidized glutathione, reduced glutathione to oxidized glutathione ratio, and uric acid [15,31,54]. Performing a submaximal cool-down immediately following a training session resulted in higher session ratings of perceived exertion [58], but post-exercise cool-down combined with cold-water immersion attenuated post-exercise impairments of agility, sprint, jump, balance, dribbling, and shooting test performance [48].

### 3.6. Blood Flow Restriction and Compression Therapy

Of the four studies investigating blood flow restriction or compression techniques, two studies revealed positive effects on jump performance, exercise-induced muscle damage markers, and perceived recovery, but two studies also found a negative impact on ratings of perceived exertion and jump performance. An active recovery session combined with blood flow restriction following a match resulted in significantly higher ratings of perceived fatigue in male youth soccer players [35]. Another study revealed minimized impairments of jump performance, muscle damage markers, and soreness following post-exercise passive blood flow restriction in semi-professional male soccer players [38].

There was no effect of wearing compression garments in the three days after a match on muscle damage markers, inflammation, and muscle soreness in semi-professional male soccer players [50], but another study with the same protocol and participant characteristics found moderate but insignificant beneficial effects for attenuating the reductions in arterial oxygenation saturation of hemoglobin, perceived recovery, and jump test performance [49].

Finally, another study compared post-exercise pneumatic cooling of the thigh to a passive recovery control group and found immediate negative effects on countermovement jump performance but no effect on hamstring flexibility or maximal isometric adductor strength in professional male soccer players [29].

### 3.7. Muscle Relaxation and Flexibility

Foam rolling after a soccer training session benefited agility test performance and perceptions of recovery and soreness but not sprint or countermovement jump performance in professional male soccer players [57] while static stretching (2 sets of 15 s stretches of the gastrocnemius, hamstrings, quadriceps, glutes, hip flexors, adductors, and abductors) immediately following a match reduced creatine kinase levels compared to a passive recovery control group in youth male soccer players [53]. No differences between the static stretching and passive recovery control group were found for countermovement jump performance, muscle swelling, or perceived muscle soreness [53].

### 3.8. Whole-Body Cryotherapy Chamber and Cooling Garments

Whole-body cryotherapy increased post-exercise testosterone responses and acutely reduced restlessness. One study found increased testosterone responses 2 and 24 h after the post-exercise use of 120 s of whole-body cryotherapy in a chamber at −135 °C in club male soccer players, but there was no effect on muscle soreness, creatine kinase, or countermovement jump performance [62]. Another study examining the post-exercise impact of whole-body cryotherapy on sleep characteristics in professional male soccer players found reduced restlessness following 180 s of cryotherapy, but there was no significant effect on other actigraphy sleep metrics [40]. Wearing lower-body garments that contained cooled phase-change material resulted in higher maximal isometric voluntary contraction and lower muscle soreness in professional male soccer players, but no effects on countermovement jump performance or mood were observed [36].

### 3.9. Mindfulness, Sleep, and Daytime Nap

Four studies investigated the impact of post-exercise sleep- or mindfulness-related interventions and found positive effects on exercise test performance, perceptions of recovery and sleepiness, nighttime sleep metrics, and cognitive performance. In two studies with male non-professional soccer players, a sleep hygiene strategy deployed after a late match significantly improved sleep quantity compared to normal sleep routines without changes in muscle damage, blood markers, or perceived recovery or stress [41] and a daytime nap opportunity improved run test performance and perceived sleepiness, and soreness [43]. In another study, sleep latency and perceived sleep quality were both improved following a post-match sleep education session compared to a control group that did not receive education, but no between-group differences were observed on the second night after the intervention [63]. Finally, a 6 min mindfulness intervention in collegiate (non-professional) male soccer players resulted in increased oxyhemoglobin concentration in the prefrontal cortex, lower cortisol levels, and improved reaction time and accuracy compared to the control group [64].

### 3.10. Nutrition

Post-exercise protein supplementation demonstrated significant beneficial effects on countermovement jump height and reactive strength index, perceived soreness, and mood [26], but there was no observed effect on glycogen resynthesis [42] or exercise-induced muscle damage markers [47]. One study demonstrated that consuming 40 g of casein after a match and before sleep was related to improved jump performance (e.g., jump height and reactive strength index), perceived soreness, and mood compared to the placebo condition in professional male soccer players [26]. In another study, post-exercise consumption of a protein- and carbohydrate-enriched diet (71%, 21%, and 8% energy from carbohydrate, protein, and fat, respectively) did not significantly affect glycogen resynthesis in professional male soccer players [42]. Deteriorations in speed-endurance performance were mitigated by increasing male soccer players’ dietary protein intake to 1.5 g/kg/day by supplementing with whey protein or soy protein, but there was no difference between the whey or soy supplementation and control groups for countermovement jump performance, creatine kinase, and muscle soreness [47].

Supplementation of beet juice [39] and curcumin [27], but not cherry juice [25] or caffeine [30], demonstrated positive effects on recovery in semi-professional, professional, and non-professional male soccer players. Chronic and post-exercise supplementation of beet juice in semi-professional male soccer players revealed benefits for countermovement and squat jump performance, sprinting, maximal voluntary contraction, and perceived muscle soreness compared to a placebo control group [39]. Similarly, post-match acute curcumin consumption attenuated decrements in countermovement jump height and reactive strength index and reduced muscle soreness compared to a placebo control in professional male soccer players [27]. Other studies have demonstrated that post-match consumption of cherry juice did not have significant effects on countermovement jump height, well-being, or soreness in professional male soccer players [25], and caffeine consumption did not significantly affect acute recovery from a shuttle run test in non-professional male soccer players [30].

## 4. Discussion

This systematic review aimed to describe the post-exercise effects of recovery strategies in male and female soccer players of all ages and competitive levels. This systematic review serves as a comprehensive resource that supports coaches, practitioners, and athletes in understanding the effects of post-exercise recovery techniques in soccer players. The main findings were as follows: (i) cold-water immersion demonstrated post-exercise improvements in jump performance and perceptions of fatigue, soreness, and overall well-being; (ii) other recovery strategies, specifically sleep interventions, nutrition supplementations (e.g., beet juice and curcumin supplementation and pre-sleep casein consumption), active recovery, and compression therapy, positively impacted recovery in soccer players, albeit with varying levels of effectiveness and evidence; (iii) not all recovery strategies that are commonly used by soccer players (e.g., pneumatic compression, massage, eletrostimulation, etc.) or that have been explored in other athletic populations (e.g., photobiomodulation, floatation-restricted environmental stimulation therapy) were investigated by studies that met the eligibility criteria for this review; (iv) few studies presented evidence of negative effects of post-exercise recovery techniques on recovery in soccer players; however, it is still critically important to consider individual’s training context, beliefs, and preferences for advising an athlete on the appropriate recovery intervention and for analyzing its effects.

The studies identified in this review demonstrated a beneficial post-exercise effect of cold-water immersion on jump test (e.g., squat jump, countermovement jump) performance [33,34,46,51] and perceived soreness, fatigue, and overall well-being [32,45,52,59]. These findings agree with previous research that describes the effects of cold-water immersion on physical performance [23,65,66,67]. A recent meta-analytical review demonstrated a mean improvement in jump performance 24 h after post-exercise cold-water immersion and mean improvements in perceived total quality recovery, fatigue, and soreness at various [65,68]. However, recent systematic reviews also recognized contradictory findings across studies as well as high potential for bias and heterogeneity [66,67,69,70,71]. For example, most cold-water immersion protocols deployed by the studies selected for the current review included water temperature at or around 10 °C, while Machado and colleagues recommend 11–15 °C over 11–15 min to be the optimal dosage to obtain recovery benefits [71]. Furthermore, findings from another review found that only immersion in water below 15 °C had positive impacts on inflammation [72]. Proposed mechanisms for the effects of cold-water immersion on post-exercise recovery include the interaction of several different pathways, such as eliciting peripheral vasoconstriction and lowering tissue metabolism to minimize inflammatory responses, and facilitating parasympathetic reactivation by increasing vagal tone and restoring heart rate variability to counteract sympathetic nervous system activity during exercise [65,68]. There is also limited and inconsistent evidence suggesting that cold-water immersion aids the clearance of exercise-induced muscle damage markers and metabolic by-products through vasoconstriction followed by vasodilation [73], which was also evident in the findings of the studies selected for the current review [32,33,34,51].

Sleep is commonly described as a fundamental for optimal recovery and a key factor for sports performance [3]. However, only three studies selected for the current review investigated the post-exercise effects of a sleep-related intervention on recovery in soccer players [41,43,63]. A recent review of the effects of napping on athletic performance revealed beneficial effects of a daytime nap opportunity on physical and cognitive performance [74], which is consistent with the findings of the study examining the impact of a post-exercise nap in soccer players [43]. Naps and extending time in bed are two of the most commonly deployed sleep interventions in athletes [74], but much of the literature suggesting the importance of sleep on athletic performance has focused on how sleep deprivation affects recovery and performance [75]. A recent review included 69 publications and found an average impact of −7.56% of sleep loss on seven categories of physical performance [75]. Furthermore, Bonnar and colleagues reported that benefits to recovery from sleeping strategies are observed after long-term interventions [76]. Thus, while the studies selected for this review demonstrate acute benefits of sleep extension and education in soccer players consistent with limited previous sleep extension research, long-term adherence to optimal sleep hygiene practices may offer greater overall benefits for recovery [76].

Multiple post-match nutrition interventions, specifically supplementation with curcumin, beetroot juice, and pre-sleep casein protein, demonstrated positive effects on recovery [26,27,39]. While post-exercise beetroot supplementation has shown mixed results in athletic populations, with some studies reporting benefits [77,78,79,80] and others finding no effect [81,82], its potential benefits are attributed to nitrate, a precursor for nitric oxide, and other bioactive compounds such as betalain and polyphenols, which can reduce inflammation [83]. Similarly, curcumin supplementation has shown positive effects on exercise-induced muscle damage markers and inflammation via ingredients that potentially suppress proinflammatory cytokines [84], despite positive but insignificant findings in a recent meta-analytical review [85]. Additionally, post-exercise and pre-sleep consumption of 40 g of casein protein has been associated with improved recovery, as evidenced by enhanced jump performance and reduced delayed onset muscle soreness in soccer players [26]. This aligns with findings in various populations, attributed to the slow increase in plasma amino acid concentrations following casein ingestion, facilitating sustained protein anabolism [86,87].

The studies identified in this review reported positive effects of post-exercise active recovery, compression garments, foam rolling, stretching, and blood-flow restriction on recovery in soccer players, albeit in specific contexts and with limited evidence. For example, one study found that blood flow restriction while lying passively in a supine position attenuated post-exercise impairments of jump performance, muscle damage markers, and soreness [38], but another study found that blood flow restriction combined with a submaximal active recovery session increased ratings of perceived fatigue [35]. Similarly, a recent systematic review on the effects of blood flow restriction on recovery reported some studies showing beneficial effects on recovery, while other studies showed no significant impact or negative effects [88]. Furthermore, following an active recovery intervention, male players experienced improvements in perceived recovery and jump test performance [54,55,56], no significant effects were observed in female professional and semi-professional players [15,52], and exercise-induced muscle damage markers remained unaffected across all studies [15,31,54]. Similar findings have been reported in other populations as well. For example, positive effects on psychological outcomes, but not blood lactate clearance rate, were observed following active recovery in professional, collegiate, and competitive adult athletes [89]. The discrepancies in findings can be attributed to heterogeneous methods, inconsistent dependent variables to quantify recovery (e.g., jump test performance, perceived soreness, creatine kinase levels, sprint performance), and varying recovery intervention protocols.

Another main finding of the present review is that recovery techniques that are commonly used among soccer players were not represented by the studies identified in the current review. For example, recent surveys of professional soccer teams reported that massage, intermittent pneumatic compression, and electrostimulation are frequently used after training or competitions [90,91]. While not represented in these recent recovery modality surveys [90,91], other recovery techniques like photobiomodulation, sauna, hyperbaric chambers, and floatation-restricted environmental stimulation therapy were also not represented in the studies identified by the current review but have been researched in other athletic and healthy populations For example, a randomized controlled trial with healthy adult males found that post-exercise targeted photobiomodulation resulted in increased maximum voluntary contraction, decreased muscle soreness, and minimized creatine kinase activity from 24 to 96 h post-exercise compared to a placebo condition [92]. However, Driller and Lebeater recently reported that these recovery techniques lack a high level of research-grade evidence, indicating that further research on the effects of these strategies in general athletic populations and in soccer players is warranted to support general and sport-specific interventions [3].

Female soccer players were significantly underrepresented in the studies identified for this review. Specifically, only 55 (8.03%) of the 685 total participants across all studies were female [15,31,61]. The low proportion of female athlete participants in the review is a major limitation but is consistent with recent audits of female athlete representation in sports medicine and sport science research [93]. Previous literature has highlighted major differences in metabolic, inflammatory, and thermoregulatory responses to exercise between male and female athletes [94]. Thus, it is inappropriate to assume that outcomes observed in the studies with exclusively male soccer players will directly translate to female soccer players. This highlights a current limitation of recovery science literature and underscores the need for research that includes female athlete participants across different ages and competitive levels. At present, the underrepresentation of female athletes means that recommendations for recovery strategies from coaches and practitioners are often extrapolated from studies conducted on male athletes, which may not accurately capture the unique physiological, hormonal, and contextual demands of female athletes and thus may not provide the best support for their health and performance. Furthermore, the demands of soccer change across age groups and competitive levels, so additional research should explore how recovery strategy recommendations should also change across ages and competitive levels to provide targeted support for athletes.

Beyond sex-based differences, competitive level and age for both male and female athletes further shape the physiological demands placed on athletes and influence how recovery strategies should be prescribed. Studies included in the final review involved varying populations, but there were no comparisons of responses to post-exercise recovery strategies between different competitive levels and ages. For instance, the testosterone response elicited by post-exercise whole-body cryotherapy was only explored in male academy soccer players 18 (standard deviation = 2) [62]. However, testosterone responses to exercise are known to change across physical maturation status in men, with higher acute values in younger males compared to older men [95]. In this example, additional investigations are warranted to explore whether the testosterone response to post-exercise whole-body cryotherapy would also be higher in young males and lower in older male athletes. Furthermore, while the studies consolidated in this review provide an aggregation of the current body of randomized controlled and randomized within-subjects crossover controlled studies exploring the effects of post-exercise recovery strategies in soccer players for coaches, practitioners, and athletes, another finding from this review is a need for additional investigations that explore the differences in responses between athletes of different ages and competitive levels at large.

Group-level analyses of the studies selected for this review revealed beneficial, unclear, and even negative impacts with varying levels of statistical evidence describing the effects of post-exercise recovery interventions. However, these statistical approaches may have overlooked significant intersubject differences in responses to recovery strategies [96,97]. An athlete’s specific needs, beliefs, and preferences are relevant for informing which recovery technique is most appropriate for restoring performance [97]. An athlete’s specific needs, beliefs, and preferences are relevant for informing which recovery technique is most appropriate for restoring performance. In practice, this should involve onboarding procedures to capture each athlete’s attitudes, beliefs, and established routines for post-training and post-match recovery. Subsequently, interventions tailored to each individual can be deployed by overlapping what an athlete is already familiar with and what is most optimal based on the context, and what is described in the literature (Table 3). Additionally, this process should be iterative as athletes are constantly redefining their attitudes and beliefs as they are exposed to new information.

While this review provides practical, evidence-informed guidance to practitioners, coaches, and athletes, including specific post-exercise recovery strategy protocols and their observed effects across studies involving soccer players of different characteristics, it is not presented without limitations. For example, this systematic review did not involve a meta-analytical review process and therefore did not present effect sizes for each recovery strategy. However, as more literature is available describing each recovery strategy, future research may involve the meta-analytical review process and subsequent prioritization of recovery strategy recommendations based on the observed effects instead of solely based on their representation in the literature. Additionally, based on practical constraints, a single reviewer screened articles against the inclusion and exclusion criteria with assistance from a co-author when there were uncertainties, conducted data extraction, and conducted the quality assessment.

## 5. Conclusions

This review demonstrated that post-exercise recovery techniques positively impact psychophysiological recovery, albeit with varying levels of consistency and evidence. It serves as a valuable resource for understanding the impacts of common post-exercise recovery techniques specific to soccer athletes, providing insights to aid coaches, practitioners, and players in making informed decisions regarding recovery strategies. Cold-water immersion (10–15 °C for 10–15 min) specifically improved jump test performance and reduced perceived soreness, fatigue, and overall discomfort, consistent with previous studies in other populations. Sleep (e.g., daytime nap and sleep extension) and nutritional interventions (e.g., beet juice, curcumin, and pre-sleep casein supplementation) also resulted in improved recovery outcomes, but long-term optimal sleep hygiene (e.g., consistent sleep quantity and quality) should be prioritized over the use of acute sleep extension for recovery. A summary of the recommended recovery interventions and their effects, informed by the studies identified for this review, is presented in Table 3. Additional recovery strategies have been studied in other athletic populations and even reportedly used by soccer players (e.g., massage, sauna, hyperbaric chamber) but were not represented by the studies selected for this review. Although these recovery techniques may still benefit soccer players, further research in soccer-specific contexts, especially in female athletes, is necessary to enhance customization and sport-specific recovery approaches. However, an individual athlete’s preferences, beliefs, training contexts, competition level, and recovery needs should also be carefully considered by practitioners before implementing recovery interventions. To account for these interindividual differences, future research and applied athlete monitoring practices should aim to implement effective data collection strategies that allow for repeated measures and replication on an individual level with recovery outcome measures and protocols that are specific and sensitive to the population under study.

## Figures and Tables

**Table 1:** Search strategy and terms, i.e., (1 AND 2) AND (3 OR 4 OR 5).

Item	Desired Outcome	Search Terms
1	Sample of soccer players	soccer OR football NOT American football
2	Recovery modality use	Recovery strategies OR recovery modalities OR pneumatic compression OR electrical stimulation OR cold water immersion OR hot water immersion OR contrast bath OR floatation-restricted environmental stimulation therapy OR photobiomodulation OR blood flow restriction OR occlusion OR cryotherapy OR active recovery OR sleep OR meditation OR mindfulness OR imagery OR nutrition OR hydration OR compression garments OR massage OR sauna OR percussive OR stretching OR yoga OR progressive muscle relaxation OR acupuncture OR pool-based recovery OR external cold OR grounding OR Earthing OR hydrotherapy OR chamber OR whole-body vibration OR breathwork protein or carbohydrate or supplement or diet or hydration or nutrition or whey OR cherry juice
3	Physical/physiological recovery	physical recovery OR muscle recovery OR neuromuscular fatigue OR fitness OR strength OR fatigue OR glycogen OR inflammation OR inflammatory markers OR neuromuscular markers OR physiological markers OR biomarkers OR muscle damage OR soreness OR pain OR physical performance OR cardiovascular recovery OR range of motion OR biochemical markers OR speed OR heart rate OR tendon damage
4	Psychological/cognitive/mental recovery	mental fatigue OR cognitive fatigue OR reaction time OR response time OR decision-making OR perceived recovery OR wellness OR stress OR rating perceived exertion OR quality recovery OR sleep OR mood OR motivation OR energy
5	Technical performance recovery	passing OR juggling OR shooting OR finishing OR dribbling

**Table 2:** Methodology assessment results.

Reference	EC	RA	CA	BL	SB	BT	BA	MA	IT	BG	PM	Total Score
Abbott et al., 2020 [25]		*	*	*	*	*		*	*	*	*	9
Abbott et al., 2019 [26]		*	*	*			*	*	*	*	*	8
Abbott et al., 2023 [27]		*	*	*	*	*	*	*	*	*	*	10
Alexander et al., 2022 [28]		*		*				*	*	*	*	6
Alexander, Keegan, Carling, and Rhodes, 2022 [29]	*	*		*				*	*	*	*	7
Andrade et al., 2015 [30]		*		*	*	*				*	*	7
Andersson et al., 2008 [15]	*	*		*				*	*	*	*	7
Andersson et al., 2010 [31]		*		*				*	*	*	*	7
Ascensao et al., 2011 [32]		*		*				*	*	*	*	6
Bouchiba et al., 2022 [33]		*		*				*	*	*	*	6
Bouzid et al., 2018 [34]		*		*				*	*	*	*	6
Castilla-Lopez & Romero-Franco, 2023 [35]	*	*		*				*	*	*	*	7
Clifford et al., 2018 [36]		*	*	*			*	*	*	*	*	8
Coelho et al., 2020 [37]	*	*		*				*	*	*	*	7
Daab et al., 2021a [38]		*	*	*		*		*	*	*	*	8
Daab et al., 2021b [39]		*		*	*	*	*	*	*	*	*	9
Douzi et al., 2019 [40]		*		*				*	*	*	*	6
Fullagar et al., 2016 [41]	*	*								*	*	4
Gunnarsson et al., 2013 [42]		*								*	*	3
Hsouna et al., 2022 [43]	*	*		*				*	*	*	*	7
Kim & Joo, 2023 [44]	*	*		*				*	*	*	*	7
Kinugasa & Kilding, 2009 [45]	*	*		*				*	*	*	*	7
Kositsky & Avela, 2020 [46]		*		*				*	*	*	*	6
Kritikos et al., 2021 [47]	*	*	*	*	*	*		*	*			8
Lee et al., 2021 [48]	*	*		*				*	*	*	*	7
Marques-Jimenez et al., 2018a [49]	*	*		*				*	*	*	*	7
Marques-Jimenez et al., 2018b [50]	*	*		*				*	*	*	*	7
Nasser et al., 2023 [51]	*	*		*	*			*	*	*	*	8
Pesenti et al., 2020 [52]	*	*	*	*		*		*	*	*	*	9
Pooley et al., 2017 [53]	*	*		*				*	*	*	*	7
Pooley et al., 2020 [54]	*	*		*				*	*	*	*	7
Rey et al., 2012a [55]		*		*				*	*	*	*	6
Rey et al., 2012b [56]		*		*				*	*	*	*	6
Rey et al., 2019 [57]	*	*		*				*	*	*	*	7
Rodríguez-Marroyo et al., 2021 [58]		*		*				*	*	*	*	6
Roswell et al., 2009 [59]		*		*				*	*	*	*	6
Roswell et al., 2011 [60]		*		*				*	*	*	*	6
Rupp et al., 2012 [61]	*	*	*	*			*	*	*	*	*	9
Russell et al., 2017 [62]		*		*				*	*	*	*	6
Vitale et al., 2019 [63]		*		*				*	*	*	*	6
Zhu et al., 2021 [64]	*	*	*	*	*			*	*	*	*	9

Abbreviations: BA, there was blinding of all assessors who measured at least one key outcome; BG, the results of between-group statistical comparisons were reported for at least one key outcome; BL, groups were similar at baseline; BT, there was blinding of all therapists/researchers who administered the therapy/protocol; CA, concealed allocation; EC, eligibility criteria were specified; PM, the study provided both point measures and measures of variability for at least one key outcome; IT, all subjects for whom outcome measures were available received the treatment or control condition as allocated or, where this was not the case, data for at least one key outcome were analyzed by “intention to treat”; MA, measures of at least one key outcome were obtained from more than 85% of the subjects initially allocated to groups; RA, subjects were randomly allocated to groups (in a crossover study, subjects were randomly allocated an order in which treatments were received); SB, there was blinding of all subjects; *, reflects the condition was satisfied while a blank cell means it was not met.

**Table 3:** Recommended recovery interventions and protocols.

Recovery Intervention		Protocol
Study Count	Recovery Strategy	
12	Cold-Water Immersion	10 to 15 °C (50 to 59 °F) cold-water immersion for 10–15 min applied within 1 h post-training/match and can be repeated on subsequent days
1	Contrast-Water Immersion	Alternate between 1 and 2 min of hot- (36 °C–40 °C or 96.8 °F–104 °F) and 1 min of cold-water (10–15 °C or 50 °F–59 °F) lower-body immersion in a pool 3 to 7 times for a total of 6–15 min applied within 1 h post-training/match and can be repeated on subsequent days
6	Active Recovery	10–20 total minutes of low-intensity cycling or running combined with dynamic stretching 24 h after training or match
2	Blood Flow Restriction	3 cycles of 5 min occlusion (50 mmHg above systolic blood pressure) separated by 5 min of reperfusion (0 mmHg) using a blood pressure cuff placed bilaterally on the proximal portion of the thigh while in a passive, supine position
1	Foam Rolling	45 s of foam rolling quadriceps, hamstrings, adductors, glutes, and gastrocnemius, beginning at the distal portion of the muscle on both legs, applied within 1 h post-training/match, and can be repeated at subsequent timepoints post-training/match
1	Static Stretching	30 s stretching quadriceps, hamstrings, adductors, glutes, and gastrocnemius within 30 min post-training/match
2	Whole-Body Cryotherapy	120–180 s at −135 °C to −180 °C applied within 1 h post-training/match and can to be repeated at subsequent timepoints post-training/match
1	Mindfulness Intervention	8 min mindfulness induction including mindful breathing (2 min mindful breathing, 4 min body scan, and then 2 min mindful breathing)
3	Daytime Nap	40 min nap opportunity in the early afternoon
1	Pre-Sleep Casein Consumption	40 g of casein within 30 min of going to sleep
1	Beet Root Juice	Consume 150 mL twice per day (08:00 AM and 6:00 PM)
1	Curcumin Supplementation	Consume 500 mg curcumin supplement within 1 h post-training/match

Abbreviations: C, Celsius; F, Fahrenheit; mmHg, millimeters of Mercury; mL, milliliters; mg, milligrams.

## Supplementary Materials

The following supporting information can be downloaded at: https://www.mdpi.com/article/10.3390/sports13100343/s1, Table S1: Characteristics of included studies for professional male soccer players. Table S2: Characteristics of included studies for semi-professional, amateur, collegiate, and non-professional male soccer players, Table S3: Characteristics of included studies for youth academy male soccer players, Table S4: Characteristics of included studies for female soccer players.
