# Supplementary material for: Post-Exercise Recovery Modalities in Male and Female Soccer Players of All Ages and Competitive Levels: A Systematic Review
Source: Sports (Basel). 2025 Oct 2;13(10):343. doi: 10.3390/sports13100343 (PMC12567902; doi:10.3390/sports13100343)
Supplement: Supplementary file 1 [file sports-13-00343-s001.zip › sports-3841403-supplementary.pdf]

sports

MDPI

63Systematic Review

Post-Exercise Recovery Modalities In Male And Female Soccer Players Of All Ages And Competitive Levels: A Systematic Review

Emaly Vatne [1,2,*], Jose M. Oliva-Lozano [2], Catherine Saenz [3], Rick Cost [2], and Josh Hagen [2,*]

[1] Human Performance Collaborative, The Ohio State University, Columbus, OH, United States
[2] United States Soccer Federation. Chicago, IL, United States; jlozano@ussoccer.org
[3] College of Education and Human Ecology, The Ohio State University, Columbus, OH, United States; saenz.11@osu.edu

* Correspondence: vatne.1@osu.edu

Academic Editor: Firstname Lastname

Received: date
Revised: date
Accepted: date
Published: date

Citation: To be added by editorial staff during production.

Copyright: © 2025 by the authors. Submitted for possible open access publication under the terms and conditions of the Creative Commons Attribution (CC BY) license (https://creativecommons.org/licenses/by/4.0/).

Table 1. Characteristics of included studies for professional male soccer players.

	Participants	Methods					Outcomes	
Reference	Sample Size Level Age (Years; Mean ± SD)	Recovery Method	Recovery Protocol	Control Group Protocol	Fatiguing Exercise	Testing Time Point(s)	Measures	Results
Rey et al., 2012a) [1]	N = 31 (male) Professional 23.5 ± 3.4	Active recovery	Low-intensity exercise and static stretching performed immediately after exercise	Sitting for duration of active recovery	Training session	Immediately before and 24 hours after the training session	Tensiomyography on the rectus femoris and biceps femoris and perceived muscle soreness	No significant differences for any of the tensiomyography parameters between the baseline and the posttest for active and control group. Active recovery minimized increase in perceived soreness between baseline and 24-hours post-training
Rey et al., 2012b) [2]	N = 31 (male) Professional 23.5 ± 3.4	Active recovery	Low-intensity exercise and static stretching performed immediately after exercise	Sitting for duration of active recovery	Training session	Immediately before and 24 hours after the training session	Countermovement jump without arm swing, 20-meter sprint, and the Balsom agility test (Balsom, 1994)	Post-training countermovement jump height was significantly greater for the active recovery group than in the control group. No other effect of recovery intervention on performance measures

 https://doi.org/10.3390/xxxxx

Kositsky & Avela, 2020 [3]	N = 10 (male) Professional Range: 18-20	Cold water immersion	20 minutes of cold-water immersion (10 ± 0.5°C)	The control group sat with their lower legs in an empty bucket for 20 minutes	Resistance training protocol	Before the exercise protocol, immediately after, and at 24 and 48 hours post exercise	Drop jumps, blood draw for measurement of creatine kinase, and perceived muscle soreness	Only the cold-water immersion group demonstrated significant signs of recovery at both 24- and 48-hours post-test for drop jump rebound jump height<br>No significant difference in increase of creatine kinase between groups<br>No significant difference between groups for muscle soreness
Bouzid et al., 2018 [4]	N = 8 (male) Professional 19.63 ± 0.74	Cold water immersion	10 minutes of cold-water immersion (10±1°C) within 10 minutes of completing the exercise protocol	Control group that completed 10 minutes of thermos-neutral water immersion (28±1 °C)	Intermittent running test	Baseline, after the recovery protocol, and 24, 48, ad 72 hours after the exercise and recovery protocol	Squat jump without arm swing, countermovement jump without arm swing, maximal voluntary contraction of the quadriceps, creatine kinase levels, and 20-meter sprint test	Cold water immersion group experienced squat jump and countermovement jump performance return to baseline faster than the thermoneutral control group<br>Sprint performance decreased at 24 hours and 48 hours post with thermoneutral control group and only at 24 hours post with cold water immersion<br>Maximum voluntary contraction and creatine kinase levels returned to baseline at 48 hours post-test in the cold

								water immersion group while they returned baseline at 72 hours post-test in the thermoneutral group
Alexander, Keegan, Carling, & Rhodes, 2022 [5]	N = 24 (male) Professional 20.58 ± 2.55	Cold water immersion (Recovery-Tub Solo)	11 minutes of cold-water immersion with target temperature of 10°C	Passive recovery which consisted of laying still in a semi-recumbent position for 11 minutes after training session	Training session	Immediately pre-training, post-training, post-intervention, and 24-hours post-intervention	Eccentric hamstring strength, isometric adductor strength, hamstring flexibility, hamstring and adductor skin surface temperature, and overall perceived wellbeing	Peak eccentric hamstring force was significantly greater for cold water immersion than passive recovery<br>Significantly greater recovery in peak torque and force for cold water immersion group than control<br>Overall psychological wellbeing scores were significantly worse at the 24-hour post-training time point for passive recovery compared to cold water immersion
Clifford et al., 2018 [6]	N = 11 (male) Professional 19±1	Cooling garments	Phase change material cooled lower body garments (15°C) worn for 3 hours post-match	Ambient lower body garments (22°C) worn for 3 hours post-match	Soccer match	Before and 12, 36, and 60 hours after the match	Mood, muscle soreness, countermovement jump height, and maximal isometric voluntary contraction	Maximal isometric voluntary contraction was higher at 36- and 60-hours post-match and muscle soreness was lower at 60 hours post-match in cold group compared to control<br>No differences between groups for

								countermovement jump performance or mood
Rey et al., 2019 [7]	N = 18 (male) Professional 20 ± 2	Foam Rolling	Foam rolling targeting their quadriceps, hamstrings, adductors, glutes, and gastrocnemius for two 45-second bouts each muscle with a 15-second rest	Passive recovery which included sitting on a bench for 20 minutes	Training session	Immediately before and 24 hours after the training session	Countermovement jump without arm swing, 5- and 10-meter sprints, T-test, sit-and-reach flexibility test, overall recovery, and perceived muscle soreness	No significant difference between groups for decrease in countermovement jump performance<br>Sprint tests were not significantly different between groups or at any time point<br>T-test performance was impaired at post-intervention only in the passive recovery group<br>Overall recovery and muscle soreness was significantly more impaired 24 hours after the training session in the passive recovery group compared to foam rolling
Abbott et al., 2020 [8]	N = 10 (male) Professional 19 ± 1	Nutrition	2 30-mL servings of tart cherry juice	Isocaloric cherry-flavored control drink	Soccer match	Before and 12, 36, and 60 hours after each match	Countermovement jump height and reactive strength index, muscle soreness, and subjective wellbeing	No differences between cherry juice and control groups at any time point
Abbott et al., 2019 [9]	N = 10 (male)	Nutrition	40 grams of casein protein post-	Control supplement	Soccer match	Baseline, and 12-36-, and	Muscle soreness, countermovement jump height and	Beneficial effects of casein protein compared to control for countermovement jump

	Professional 19 ± 1		exercise and 30 minutes before going to bed			60-hours post-match	reactive strength index, and mood	height and reactive strength index, mood, and soreness at 12- and 36-hours post-match
Abbott et al., 2023 [10]	N = 11 (male) Professional 19 ± 1	Nutrition	500 mg/d of a curcumin supplement	1000 mg/d of medium chain triglyceride oil control	Soccer match	Baseline, and 12- 36-, and 60-hours post-match	Muscle soreness, countermovement jump height and reactive strength index, and subjective well-being	Attenuated deficits for countermovement jump height and reactive strength index and muscle soreness, and wellbeing in curcumin group compared to control across post-match timepoints
Gunnarson et al., 2013 [11]	N = 16 (male) Professional 24 ± 1	Nutrition	High- carbohydrate and whey protein diet 48 hours post-match	Normal diet 48 hours post-match	Soccer match or simulated soccer match	Before and after the match and 24- and 48-hours post-match	Muscle biopsy and blood sample to measure creatine kinase and plasma myoglobin	No differences between groups for glycogen resynthesis and glycogen was lower in type II muscle fibers in the high-carbohydrate and protein group at 48-hours post-match<br>Plasma myoglobin was lower after the match and creatine kinase was higher 24 hours post-match in high-carbohydrate and protein group compared to control

Kritikos et al., 2021 [12]	N = 10 (male) Professional 21 ± 1.5	Nutrition	Whey protein isolate supplement group or soy protein isolate supplement group	Iso-energetic placebo control drink	Speed-endurance training sessions	1, 2, 3, 24, and 48 hours after the training session (blood sampling and soreness only at 48 hours)	Isokinetic and maximal isometric strength, 10- and 30-meter sprint, countermovement jump, repeated sprint ability, blood lactate concentrations, and muscle soreness	10-meter sprint performance decrements were significantly minimized in the whey and soy groups compared to control<br>Attenuated reductions in speed endurance for whey and soy protein groups compared to control<br>No other differences between groups
Douzi et al., 2019 [13]	N = 9 (male) Professional 24.8±5.5	Whole-body cryotherapy chamber (Cryotechno®, Castelnau le Lez, France)	Cryotherapy at −180°C for different durations: 180-second exposure, 2 90-second exposure separated by a 5-min rest at room temperature,<br>and 90-second exposure	Control group that received no cryostimulation	Training session	After recovery intervention and the night following the recovery intervention	Skin temperature of quadriceps, perceived thermal sensation, subjective sleep quality, and wrist actigraphy to monitor sleep	Significantly greater decrease in skin temperature and increase in perception of cold was seen after 180 seconds of exposure compared to other durations<br>The number of movements during the night after partial-body cryostimulation was significantly reduced and sleep quality was improved only in the 180-second exposure condition compared to control group and other tested conditions

Table 2. Characteristics of included studies for semi-professional, amateur, collegiate, and non-professional male soccer players.

	**Participants**	**Methods**					**Outcomes**	
Reference	Sample Size Level Age (Years; Mean ± SD)	Recovery Method	Recovery Protocol	Control Group Protocol	Fatiguing Exercise	Testing Time Point(s)	Measures	Results
Daab et al., 2021 [14]	N = 12 (male) Semi-professional 23 ± 1	Blood flow restriction	3 cycles of 5 minutes of partial occlusion and 5 minutes of reperfusion using a blood pressure cuff	Same blood flow restriction protocol but with reduced pressure compared to experimental group	Simulated soccer intermittent running test	Immediately after test and recovery intervention, and at 24-, 48-, and 72-hours after the test and recovery intervention	Maximal voluntary contraction of quadriceps, squat jump, countermovement jump, 20-m sprint, blood sample analysis to measure creatine kinase activity, lactate dehydrogenase, and serum C-reactive protein, and perceived muscle soreness	Blood flow restriction group attenuated decreases in squat jump countermovement jump ability 24-hours and 48-hours post-exercise compared to control<br>Maximal voluntary contraction recovered significantly greater in blood flow restriction group at 4- and 48-hours post-test<br>Muscle soreness was significantly lower immediately after and 24 hours post-test in the blood flow restriction group<br>Creatine kinase and lactate dehydrogenase were

								significantly lower in blood flow restriction group compared to control group 24-hours post-exercise
Nasser et al., 2023 [15]	N = 12 (male) Semi-professional 21.1 ± 2.2	Cold water immersion	15 minutes of cold-water immersion (11°C)	Passive recovery protocol was not described; placebo group consumed a placebo beverage	Simulated soccer intermittent running test	Before the exercise protocol, and 24 and 48 hours after the test and recovery protocol	Creatine kinase, C-reactive protein, uric acid, squat jump, countermovement jump, 10-meter sprint, 20-meter sprint, repeated sprint ability, and perceived muscle soreness	No difference between groups across timepoints for creatine kinase, but C-reactive protein was high at 24 hours post-test only in the cold-water immersion and passive recovery conditions<br>Uric acid was higher for only the rest condition at 24 and 48 hours post compared to baseline<br>Squat and countermovement jump performance decreased significantly at each time point compared to baseline in the rest condition but not in placebo or cold-water immersion group<br>Muscle soreness was higher at 24 hours post-

								test compared to baseline for only the rest condition
Bouchiba et al., 2022 [16]	N = 12 (male) Semi-professional 22.9 ± 0.9	Cold water immersion	10 minutes of cold-water immersion (10 ± 2°C)	10 minutes of thermoneutral-water immersion (28 ± 2°C)	Simulated soccer intermittent running test	After recovery protocol, and 24, 48, and 72 hours after the simulated match-play	Maximum voluntary isometric contractions of the quadriceps, electromyography, squat jump, countermovement jump, 20-meter sprint, and venous blood samples to measure plasma creatine kinase, and lactate dehydrogenase	The decrease in maximum voluntary contraction, voluntary activation, and quadriceps resting twitch force was minimized in the cold-water immersion group compared thermoneutral water control group and returned to baseline at 24 hours in the cold water immersion group and were more impaired and returned to baseline in 48-72 hours in the control group<br>Squat jump performance and plasma lactate dehydrogenase were less impaired following cold water immersion than in thermoneutral water immersion
Pesenti et al., 2020 [17]	N = 28 (male) Semi-professional	Cold water immersion (Cryo Control –	Cold water immersion for 10 minutes	Control group sat in a chair for	Fatiguing leg	24, 48, and 72 hours after the	Electromyography of the vastus medialis oblique, vastus lateralis,	No significant differences between groups for muscle recruitment or postural control

	Range: 16-19	Ice Bath Systems) and active recovery	(10°C) or walking on a treadmill for 10 minutes	10 minutes	extension protocol	exercise protocol	and rectus femoris muscles in maximum isometric contraction, postural stability, and mediolateral directions and perceived ratings of muscle soreness and pain intensity	Pain intensity in the cold-water immersion group returned to baseline after 72 hours, while the other groups continued to feel elevated pain
Coelho et al., 2020 [18]	N = 25 (male) Collegiate 21.8 ± 3.2	Cold water immersion and far-infrared emitting ceramic materials	10 minutes of cold-water immersion (10°C) for cold water immersion group and ceramic group wore far-infrared emitting ceramic materials pants during two	Control group remained seated for 30 minutes following the completion of the match in a controlled environment	Soccer match	Before and after recovery protocol, and 24 and 48 hours after the match	Blood samples to analyze creatine kinase activity, superoxide dismutase, thiobarbituric acid reactive species, and lactate dehydrogenase activity, countermovement and squat jumps, 20-meter sprint test, and perceived muscle soreness	All measures reflected impairments post-match, but there were no significant differences between conditions<br>Increases in perceived muscle soreness observed post-match but no differences between groups

			overnight sleep periods (~8 h each)					
Kim & Joo, 2023 [19]	N = 11 (male) Semi-professional 25.4 ± 5.2	Cold-water immersion and cold-water immersion combined with protein supplem-entation	10 minutes of cold-water immersion (8°C). 10 minutes of cold water immersion (8°C) combined with 1.8 g/kg of powdered protein immediately after exercise	Control group sat passively in a chair for 10 minutes at room temperature	Resistance training protocol	Immediately before the exercise protocol, immediately after the exercise protocol, and then 6, 24, 48 hours and 7 days after the exercise protocol	Muscle strength measured with bilateral isometric contractions, 40-meter sprint test, vertical jump without arm swing, and subjective measures of muscle soreness and recovery	Muscular strength was not different between recovery conditions at any time point<br>The 40-meter sprint time was significantly slower in the protein and cold-water immersion group at 6 hours post exercise<br>Vertical jump height was never impaired in protein and cold-water immersion group compared to baseline<br>Perceived soreness was not significantly different between groups
Lee et al., 2021 [20]	N = 21 (male) Collegiate	Cool down exercise or cool down	Cool down exercise group walked for	Control group sat and rested	Soccer match	Before the soccer match	The Southeast Missouri Agility Test, 20-meter sprint test with	Only the cool down combined with cold water immersion group had comparable pre- and post-

	20.28 ± 1.11	exercise combined with cold water immersion	10 minutes and then performed free exercise, including stretching for 5 minutes. The cool down combined with cold water immersion completed the same cool down exercise and then completed 10 minutes cold-water immersion (10 °C)	for 15 minutes		and 20 hours after the recovery intervention	best of 2 trials retained, countermovement jump, Y-Balance Test, 22-meter dribble test, instep shooting speed and accuracy test	exercise Southeast Missouri Agility Test, 20-m sprint test, vertical jump test, and Y-balance scores Significant reduction pre-test to post-test for dribble test for control group and cool-down group, but not cool down combined with cold water immersion group Significant reduction pre-test to post-test shooting test for control group but not for cool-down group or cool down combined with cold water immersion group
Hsouna et al., 2022 [21]	N = 13 (male) non-professional	Daytime nap	40-minute daytime nap	Control group sat awake	Simulated soccer	20 hours post-exercise protocol	5-meter shuttle run test for best distance and total distance, rating of	Significantly greater total distance and best distance for the nap group than the

	soccer players 23 ± 3			for 40-minute window	intermittent running test	and 3 hours post-nap or control window	perceived exertion, and perceived muscle soreness and sleepiness	no nap group during the 5-meter shuttle test Sleepiness and soreness were both significantly lower in the nap group than the no nap group
Zhu et al., 2021 [22]	N = 17 (male) Collegiate 20.14 ± 1.65	Mindfulness intervention	Mindfulness intervention group received a 6-minute mindful induction including a mindful breathing and body scan conducted by a clinical psychologist	Control group consisted of a 6-minute audio file that introduced the scenery of a park	Lab-based soccer protocol	Immediately before the exercise protocol and after the exercise protocol and intervention or control	Cerebral oxygenation, salivary cortisol, Stroop Task, and Corsi block-tapping task	Increased oxyhemoglobin concentration in the prefrontal cortex coupled with lower cortisol levels following mindfulness intervention versus control Reaction time and accuracy improved pre versus post for the mindfulness intervention group
Andrade et al., 2015 [23]	N = 11 (male) non-professional 25.4 ± 2.3	Nutrition	Carbohydrate: 1.2 g·kg−1 body mass·h−1	Distilled water and cellulose	Simulated soccer	4 hours after the exercise protocol	Vertical jump test, Loughborough soccer passing test, 30-meter repeated sprint test,	No effect of supplementation group on any of the outcome measures

			carbohydrate; Caffeine: 6 mg·kg−1 body mass; (iii) carbohydrate combined with caffeine	placebo control	intermittent running test		rating of perceived exertion, and plasma lactate	
Daab et al., 2021 [14]	N = 13 (male) Semi-professional 22.12 ± 0.56	Nutrition	2 servings of 150 ml nitrate-rich beetroot juice (250 mg/d) per day	Placebo control	Simulated soccer intermittent running test	Before and after exercise, and 24-, 48-, and 72-hours post-exercise	Squat jump, countermovement jump, maximal voluntary contraction of quadriceps, 20-meter sprint test, creatine kinase, lactate dehydrogenase, C-reactive protein, and perceived muscle soreness	Attenuated decreases in countermovement, maximal voluntary contraction of quadriceps, and sprint performance in beetroot juice group<br>Lower perceived muscle soreness in beetroot juice<br>No between-group differences in any venous blood markers
Vitale et al., 2019 [24]	N = 9 (male) Non-professional 25.2 ± 6.5	Sleep hygiene education	The sleep hygiene group attended a 40–45-	Control group received no education	Training session	The night preceding the day of	Sleep monitoring using wrist actigraphy Philips Respironics, Bend, OR, USA)	Sleep latency was significantly lower for sleep hygiene group compared to control on the first night after the education

			minute sleep education session where they were instructed by a specialist in sleep research and athletic recovery	about sleep hygiene		the sleep education and training session and the two nights after	to measure sleep onset latency, sleep offset, sleep efficiency, wake after sleep onset, total sleep time, immobility time, and fragmentation index as well as subjective sleep quality	Subjective sleep quality was better for the education group than control group for the first night All values returned to baseline on the second night following the intervention or control
Fullagar et al., 2016 [25]	N = 20 (male) Semi-professional 25.2 ± 6.5	Sleep hygiene strategy	Standardized pre-sleep routine and protocol	Normal sleep behaviors	Soccer match	3 nights before and night after matches, and 12- and 36-hours post-match	Actigraphy and subjective sleep data, counter-movement jump, intermittent running test performance, creatine kinase, urea, c-reactive protein, and perceived recovery	No differences between groups for perceived stress or recovery, blood markers, countermovement or running test performance, sleep onset latency, or sleep efficiency Greater wake episodes in sleep hygiene group Maximum heart rate was significantly greater in control group compared to sleep hygiene group during running test
Marques-	N = 18 (male)	Three types of	Each condition	No compression	Soccer match	Pre-match,	Countermovement jump,	Beneficial but significant effect in experimental

Jimenez et al., 2018a [26]	Semi-professional 24.00 ± 4.07	compression garments: compression stockings, compression tights, and compression shorts	included wearing one type of compression garment during the match and 7 hours per day for 3 days post-match	garments		halftime, immediately after the match, and 24-, 48-, and 72-hours post-match	aerobic running test performance, 20-meter sprints, T-tests, perceived recovery, and blood concentrations of lactate and arterial blood oxygen saturation	groups versus control group for RPE, countermovement jump, aerobic run, and T-test performance, but no difference between groups for sprint performance, perceived recovery, or arterial blood oxygen saturation<br>Blood lactate was higher post-match in the experimental group than control
Marques-Jimenez et al., 2018b [27]	N = 18 (male) Semi-professional 24.00 ± 4.07	Three types of compression garments: compression stockings, compression tights, and compression shorts	Each condition included wearing one type of compression garment during the match and 7 hours per day for 3 days post-match	Normal garments	Soccer match	One hour before the match, halftime, immediately after the match, and 24-, 48-, and 72-hours post-match	Exercise-induced muscle damage biomarkers, and calf, hamstring, quadriceps, and tibialis soreness and swelling	Positive but insignificant attenuations of muscle damage biomarkers in experimental groups<br>Thigh swelling peaked 24 hours post-match in control group and full-leg group, and 72 hours post-match in experimental group, stockings group and shorts group<br>Calf swelling increased over time for the control group and peaked at 72 hours post-match, but decreased at each

								measurement point in the experimental groups<br>Muscle soreness peaked between post-match and 24 hours post-match for all groups

Table 3. Characteristics of included studies for youth academy male soccer players.

	Participants	Methods					Outcomes	
Reference	Sample Size Level Age (Years; Mean ± SD)	Recovery Method	Recovery Protocol	Control Group Protocol	Fatiguing Exercise	Testing Time Point(s)	Measures	Results
Pooley et al., 2020 [5]	N = 15 (male) Youth academy 16.0 ± 1.0	Active recovery and cold-water immersion	Cold water immersion consisted of 10 minutes submersion (14 ± 0.8°C) or 10 minutes low-intensity active recovery	Static stretching	Soccer matches (2 40-minute halves)	Before the match, immediately after, and 48 hours post-match for each recovery	Muscle edema, creatine kinase, countermovement jump with arm swing and perceived muscle soreness	Countermovement jump performance recovered to baseline by 48 hours post-match for active recovery and cold-water immersion groups but not control<br>Creatine kinase returned to baseline for only cold-water immersion group at 48 hours post-match but not control or active recovery

						intervention		Active recovery and cold-water immersion groups demonstrated significant improvements in perceived muscle soreness compared to control
Castilla-Lopez & Romero-Franco, 2023 [7]	N = 40 (male) Youth club soccer 17.06 ± 0.77	Blood flow restriction (Occlusion Cuff, Belfast, United Kingdom)	Active recovery session completed with partial blood flow restriction at 24 hours post-match	Same recovery session as blood flow restriction group but without the partial blood flow restriction	Soccer match	24-hours before the match, immediately after the match, and 24-, 48-, and 72-hours post-match	Countermovement jump without arm swim, rating of perceived exertion, and perceived fatigue, sleep quality, general muscle soreness, stress level and mood	No significant differences between groups in countermovement jump height Perceived fatigue was significantly greater 24 hours post-match in blood flow restriction group compared to control No difference between groups in perceived fatigue, sleep quality, general muscle soreness, stress level and mood
Roswell et al., 2011 [8]	N = 13 (male) Youth club soccer players 15.9 ± 0.6	Cold water immersion	5 alternating exposures of: 60 seconds of immersion in a cold bath (10 ± 0.5°C) and	Same protocol as cold-water immersion group but	3 Soccer matches in 4 days	Immediately after and 22 hours after each match	Time spent in low, moderate, and high heart rate zones, total distance, high intensity running distance (running speed >15	Cold water immersion reduced the decrement in total running distance from match 1 to matches 3 and 4 No other differences between treatment groups

			60 seconds seated rest on a chair at room temperature (24°C)	immersing in thermoneutral water (34 ± 0.5°C)			kilometers per hour) during the match, perceived leg soreness, and general fatigue	for match running performances were observed Cold water immersion group reported lower ratings of leg soreness and general fatigue
Roswell et al., 2009 [9]	N = 13 (male) Youth club soccer players 15.9 ± 0.6	Cold water immersion	5 rounds of alternating exposure of 60 seconds of cold-water immersion (10 ± 0.5°C) and 60 seconds seated rest at room temperature	Same protocol as cold-water immersion group but immersing in thermoneutral water (34 ± 0.5°C)	4 soccer matches in 4 days	90 minutes before each match across the 4 days and 22 hours after the final match	Countermovement jump, 20-meter sprints, and blood draw to measure interleukin-1b, interleukin-6, interleukin-10, fatty acid binding protein, creatine kinase, and myoglobin	No significant differences between groups for countermovement jump height and repeated sprint ability but all groups decreased in performance across the 4 days. Creatine kinase and lactate dehydrogenase increased comparably in both groups but there were no changes over time for the other biological markers
Ascensao et al., 2011 [11]	N = 20 (male) Academy 18 ± 0.8	Cold water immersion	10 minutes of cold-water immersion (10°C)	Control group fully immersed lower limbs in	Soccer match	Immediately before the match, after the recovery	Blood samples were collected for the analysis of myoglobin, creatine kinase, and C-reactive	Cold water immersion group had attenuated impairments in quadriceps strength performance and increase in creatine kinase, myoglobin, and C-

			water at 35°C for 10 minutes		inter-vention, and at 30 minutes, 24 hours, and 48 hours post-match	protein, counter-movement jump, 20-meter sprint, maximum voluntary isometric contraction of the quadriceps, and perceived muscle soreness	reactive protein after the match compared to control group<br>Cold water immersion group had minimized alterations compared to the control group for quadriceps, calf, and adductor soreness
Kinugasa & Kilding, 2009 [20]	N = 28 (male) Youth club soccer players 14.3 ± 0.7	Contrast water therapy and a combination of cold-water immersion with active recovery (iCool; Portacovery Australia, Canberra, Australian	Contrast water therapy (3 cycles of 1 minute in cold water (12° C) followed by a 2-minute hot shower (38° C))<br>Combination group (3 cycles of cold immersion for 1 minute	Passive recovery group included 7 minutes of static stretching and 2 minutes with their legs raised above	Soccer match	Before each match, 10 minutes after each match, after each recovery method, and 24 hours after the recovery method	Countermovement jump with arm swing, resting heart rate, and perceived recovery	There was no difference in vertical jump height after 24 hours or between the conditions<br>Perceived recovery immediately after combination recovery was significantly higher than for contrast therapy or passive recovery<br>Players perceived lighter legs in the combination group compared to contrast therapy and passive at post-24 hours

		Capital Territory, Australia)	followed by active recovery using a cycle ergometer for 2 minutes)	heart level				
Rodríguez-Marroyo et al., 2021 [21]	N = 16 (male) Youth club soccer players 15.7 ± 0.4	Cool down	5, 10, 15-minute submaximal active cool down exercises	Passive recovery which consisted of 5-minutes of lower limb static stretching	Training sessions across 6 weeks	30 minutes after the training sessions	Rating of perceived exertion	The session rating of perceived exertion was significantly greater in active recovery group than passive recovery, especially after hard training session Session RPE was lowest for the 5-minute active cool down and highest for the 15-minute active cool down
Alexander et al., 2022 [33]	N = 18 (male) Academy 18 ± 0.5	Pneumatic cooling (Game Ready)	Pneumatic cooling was applied to both lower limbs (circumferentially wrapped	Passive recovery which consisted of laying still in a semi-recumbent	Training session	Before training, immediately post-training, and immediately	Hamstring flexibility and hamstring and quadriceps skin surface using (Infrared Thermal Imaging (ThermoVision A40M, FLIR, Danderyd,	Significant reduction in CMJ performance in the group exposed to cryotherapy immediately after intervention but not in passive control Skin temperature decreased significantly in pneumatic cooling group

			around the thigh) for a 20-minute dose with medium intermittent compression	position for 20 minutes without pneumatic cooling		post-intervention	Sweden), countermovement jump performance without arm swing, and maximum isometric adductor strength	No significant changes in hamstring flexibility or isometric adductor strength was found
Russell et al., 2017 [40]	N = 14 (male) Academy 18 ± 2	Whole-body cryotherapy (BOC Cryotherapy Chamber, Linde, Surrey, United Kingdom	30 seconds in a pre-cooling chamber (-60° C) then 120 seconds in the second chamber (-135° C) and then seated for 95 minutes in the same room used for the control	The control group players remained seated in a temperate environment for 110 minutes	Simulated soccer intermittent running test	Immediately before the exercise protocol, after the exercise protocol, and 2 and 24 hours after the exercise protocol	Capillary blood and saliva samples for creatine kinase and testosterone, countermovement jump tests, and perceived muscle soreness and recovery	A greater testosterone response was observed at 2-hours and 24-hours post exercise for cryotherapy compared to control<br>No between treatment differences were observed for perceived muscle soreness and recovery, lactate, cortisol, creatine kinase, or peak power output

Table 4. Characteristics of included studies for female soccer players.

	**Participants**	**Methods**					**Outcomes**	
Reference	Sample Size Level Age (Years; Mean ± SD)	Recovery Method	Recovery Protocol	Control Group Protocol	Fatiguing Exercise	Testing Time Point(s)	Measures	Results
Andersson et al., 2010 [1]	N = 16 (female) Professional 22 ± 3	Active recovery	Low-intensity training program and low-intensity resistance training performed at 22 and 46 hours after the first match	Passive recovery regimen not described	2 90-minute soccer matches separated by 72 hours	Immediately before the games; immediately after the games; 21, 45 and 69 hours after the first game	Blood samples to analyze reduced glutathione (GSH), oxidized glutathione (GSSG) and reduced glutathione to oxidized glutathione ratio (GSH:GSSG)	The GSH and GSSG and GSH:GSSG ratio were not affected by active recovery, GSSG increased by the same extent after both games
Andersson et al., 2008 [2]	N = 17 (female) Professional 22.6 ± 4.2	Active recovery	Low-intensity training program and low-intensity	Passive recovery regime not described	90-minute soccer match	3 hours before the match, immediately	Countermovement jump without arm swing, 20-meter sprint, maximal isokinetic knee flexion	There were no significant differences in the recovery pattern between the active and passive recovery groups

			resistance training performed at 22 and 46 hours after the first match			after, and 5, 21, 27, 45, 51, 69, and 74 hours after the match	and extension, creatine kinase, urea, and uric acid, and perceived muscle soreness	
Rupp et al., 2012 [14]	N = 22 (9 female) Collegiate 19.8 ± 1.1	Cold water immersion	15 minutes cold water immersion (12°C)	Control group remained seated for 15 minutes at room temperature	Simulated soccer intermittent running test	Post-exercise, and 24 and 48 hours post exercise	Countermovement jump, simulated soccer intermittent running test, and perceived fatigue	No significant differences between the groups on intermittent running test performance<br>No significant differences between groups for countermovement jump performance<br>No significant differences between the groups in perceived fatigue
